# A fully-automatic caudate nucleus segmentation of brain MRI: Application in volumetric analysis of pediatric attention-deficit/hyperactivity disorder

**DOI:** 10.1186/1475-925X-10-105

**Published:** 2011-12-05

**Authors:** Laura Igual, Joan Carles Soliva, Antonio Hernández-Vela, Sergio Escalera, Xavier Jiménez, Oscar Vilarroya, Petia Radeva

**Affiliations:** 1 Department of Applied Mathematics and Analysis, University of Barcelona (UB). Gran Via de les Corts Catalanes 585, Barcelona (08007) Spain; 2 Computer Vision Center (CVC), Campus UAB, Edifici 0, Bellaterra, Barcelona (08193) Spain; 3 Unitat de Recerca en Neurociència Cognitiva (URNC), Department of Psychiatry, Universitat Autònoma de Barcelona (UAB), IAPS Hospital del Mar. Passeig Marítim, 25-29 Barcelona (08003) Spain; 4 Fundació IMIM. Dr. Aiguader, 88, Barcelona (08003) Spain; 5 Image Processing Department, CRC CIM (Molecular Imaging Center), Dr. Aiguader, 88, Barcelona (08003) Spain

**Keywords:** Brain caudate nucleus, segmentation, MRI, atlas-based strategy, Graph Cut framework

## Abstract

**Background:**

Accurate automatic segmentation of the caudate nucleus in magnetic resonance images (MRI) of the brain is of great interest in the analysis of developmental disorders. Segmentation methods based on a single atlas or on multiple atlases have been shown to suitably localize caudate structure. However, the atlas prior information may not represent the structure of interest correctly. It may therefore be useful to introduce a more flexible technique for accurate segmentations.

**Method:**

We present *Cau-dateCut*: a new fully-automatic method of segmenting the caudate nucleus in MRI. CaudateCut combines an atlas-based segmentation strategy with the Graph Cut energy-minimization framework. We adapt the Graph Cut model to make it suitable for segmenting small, low-contrast structures, such as the caudate nucleus, by defining new energy function data and boundary potentials. In particular, we exploit information concerning the intensity and geometry, and we add supervised energies based on contextual brain structures. Furthermore, we reinforce boundary detection using a new multi-scale edgeness measure.

**Results:**

We apply the novel CaudateCut method to the segmentation of the caudate nucleus to a new set of 39 pediatric attention-deficit/hyperactivity disorder (ADHD) patients and 40 control children, as well as to a public database of 18 subjects. We evaluate the quality of the segmentation using several volumetric and voxel by voxel measures. Our results show improved performance in terms of segmentation compared to state-of-the-art approaches, obtaining a mean overlap of 80.75%. Moreover, we present a quantitative volumetric analysis of caudate abnormalities in pediatric ADHD, the results of which show strong correlation with expert manual analysis.

**Conclusion:**

CaudateCut generates segmentation results that are comparable to gold-standard segmentations and which are reliable in the analysis of differentiating neuroanatomical abnormalities between healthy controls and pediatric ADHD.

## 1 Introduction

Studies of volumetric brain magnetic resonance imaging (MRI) show neuroanatomical abnormalities in pediatric attention-deficit/hyperactivity disorder (ADHD) [1-3]. ADHD is a developmental disorder characterized by inatten-tiveness, motor hyperactivity and impulsiveness, and it represents the most prevalent childhood psychiatric disorder. It is also estimated that half the children with ADHD will display the disorder in adulthood. As stated in several reviews and metanalyses, diminished right caudate volume is one of the most replicated findings among ADHD samples in morphometric MRI studies [4]. As a result of these studies, in [5], the authors proposed a diagnostic test based on the ratio between right caudate volume and the total bilateral caudate volume.

**Figure 1:**
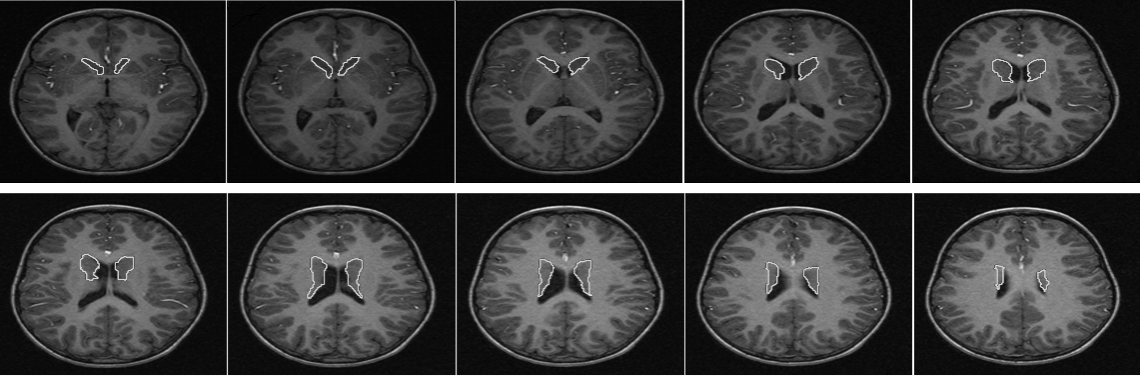
**Caudate nucleus in MRI transversal planes**. Examples of brain MRI transversal scans. Caudate nuclei are marked in white.

Most of the analyses of ADHD via MRI images, as well as much research in neuroscience, lack an appropriate automated segmentation system, and therefore require physicians to manually segment brain structures, such as the caudate, on a slice by slice basis. This process is extremely time consuming, tedious, and prone to inter-rater discrepancies, limiting the statistical power of the analysis. An automated approach would accelerate the analysis and make the procedure feasible for large amounts of data. Automatic segmentation of subcortical structures in the brain is currently an active research area. In contrast to the problem of tissue segmentation (GM, WM, and CSF) in brain MRI, for which acceptable solutions can be found, the issue of subcortical structure segmentation has yet to be satisfactorily addressed. Structures such as the putamen and caudate nucleus are difficult to correctly segment even manually, since they are small and their intensity is non-uniform and non-contrasted. Figure 1 is an example of some brain MRI transversal planes with the caudate nucleus indicated.

Semi-automatic methods for segmenting subcortical structures have been proposed, such as the method developed specifically for neuroanatomical segmentation [6], in which the user specifies two coordinates of the AC-PC line for the segmentation of the caudate. This method is a knowledge-driven two-step algorithm. In the first step, lateral ventricles are extracted to help position a bounding box that contains the caudate nucleus. Region growing of gray matter seed points is performed inside the box to estimate an initial segmentation. A set of anatomical constraints are also defined, based on previous knowledge, and are subsequently imposed on the first result. In the second step, the caudate boundaries are refined outside the bounding box by imposing new anatomical constraints. In [1], the authors use an SPM tool to segment and compute voxel-based morphometry measures. Significant effort has been put into automated segmentation of different structures in brain MRI (see reviews [7,8]). A good example of these efforts can be found in the Caudate Segmentation Evaluation challenge (CAUSE07) [9]. In this competition, different algorithms designed to segment the caudate nucleus from brain MRI scans were compared. From among the methods adopted, the atlas-based segmentation approaches stand out as a powerful generic technique for automatic delineation of structures in volumetric images. This approach uses data obtained from different subjects to construct an atlas, which acts as a common anatomy for the area imaged (brain) and applies it to further segmentations. The results of the CAUSE07 competition show that multi-atlas segmentation methods can outperform schemes based on a single atlas. However, running multiple registrations on volumetric data requires a lot of time, and it is difficult to determine the optimum number of atlases to be considered [10]. In contrast, an important disadvantage of atlas-based methods is that the target object is not necessarily correctly represented by the atlas shapes. In this case, a more flexible and adaptive technique can be useful in order to ensure accurate segmentation results.

In this work, we combine the power of atlas-based segmentation with an adaptive energy-based scheme based on the Graph Cut (GC) framework, to obtain a globally optimal segmentation of the caudate structure in MRI. The GC theory has been used in many computer vision problems [11]. In particular, it has successfully been applied to binary segmentation of images, and has yielded a solution which corresponds to the global minimum of an energy function [12,13]. The goodness of the solution depends on the suitability of the unary and boundary energy terms and their reliable computation. The original GC definition is limited to image information, and can fail when the caudate structure in MRI is subtle and contrast is low. In order to overcome this problem, we add supervised contextual information of the caudate nucleus and reinforce boundary detection using a new multi-scale edgeness measure.

Our method, *CaudateCut*, starts with an initialization step based on a standard atlas-based method, and defines a new GC energy function that is specially adapted to caudate nucleus segmentation. In particular, CaudateCut involves several stages. The first stage is devoted to defining the initial region of the caudate nucleus and does so by taking advantage of the *a priori *brain structure information. Later steps continue the definition of the novel GC energy function that is appropriate for segmentation of the caudate nucleus from brain MRI scans. More specifically, we propose a novel energy function that combines local and contextual image information analysis by modeling foreground and background properties, as well as relations between neighboring pixels. In contrast to the classical GC model, where energy unary terms are only based on pixel intensity values, we also exploit previously-learned shape relations. In particular, our unary term is defined as the weighted sum of two terms: one based on the intensity model, and the other on the confidence of the output of a binary classifier. The new supervised unary term uses correlogram structure as a pixel description in order to capture contextual intensity relations around the pixel analyzed. Moreover, in the case of the boundary term, we propose that information from the first and second intensity derivatives be considered, and include a measure of edgeness based on a new multi-scale version of the adaptive regularization parameter [14]. With this new term, we obtain a more accurate segmentation in the presence of boundary artifacts and improve boundary term pixel influence.

We present results from two different datasets. The first consists on an MRI dataset of thirty nine children/adolescents with ADHD (ages 6-18) and forty healthy control subjects matched for age, gender, and handedness. The second is a public dataset of 18 healthy controls from the Internet Brain Segmentations Repository provided by the Center for Morphometric Analysis at Massachusetts General Hospital. We show that our method, CaudateCut, improves segmentation performance with respect to a classical atlas-based approach and a multi-atlas approach proposed recently. Moreover, we provide a quantitative volumetric analysis of pediatric ADHD, and obtain specifications and results that are comparable to manual analysis based on caudate nucleus appearance.

The rest of the paper is organized as follows: Section 2 goes through the related work. Section 3 introduces the CaudateCut algorithm. Section 4 reports and discuss the results of experiments on caudate nucleus segmentation, as well as an ADHD volumetric quantitative analysis. Finally, Section 5 concludes the paper and describes future lines of research.

## 2 Related work

Different strategies can be adopted for fully-automatic segmentation of subcortical structures. Recent techniques can be summarized in four groups: a) anatomical atlas-based and multi-atlas-based algorithms, b) supervised learning techniques, c) statistical model approaches, and d) energy-based segmentation techniques.

a) Anatomical atlas-based methods rely on comparing the image under study with a pre-computed anatomical atlas of the brain. After the comparison, atlas label propagation is performed to give an estimation of the segmentation in the subject being studied [15-17]. Thus, these methods use knowledge about the structure of the brain directly. [15] develops ANIMAL, a fully-automatic procedure for segmenting any structure in an anatomical image in a predefined native space in an anatomical atlas in a normalized space. They observed that since the deformation field is bandlim-ited, irregular structures could not be accurately segmented. It was in their next work, ANIMAL+INSECT [18], that the problem was addressed by introducing post-processing that required tissue classification of the subject in order to refine the final segmentation of any labeled structure. Other authors have exploited the benefit of generative models with the aim of reaching optimal solutions. [19] and [20] combine tissue classification, bias correction, and non-linear warping within the same framework. Version 8 of SPM [21] includes the unified approach of [20]. An important disadvantage of these methods is the computational cost necessary to build an atlas from different subjects. Moreover, the training set selection required to build the atlas is a difficult issue, and most of the methods in Challenge CAUSE07 [9] select different training sets manually to segment the different groups of test data. This fact converts these methods in semi-automatic. In [17], the influence of atlas selection is analyzed by comparing the segmentation of tissue from brain MRI of young children using different atlases. In this case, a standard expectation-maximization algorithm with registration-based segmentation was used [22]. In [23], the authors incorporate structure-specific models using Markov random fields and [24] improves the results produced by [23] using diffeomorphic warps.

Atlas-based algorithms were first based on a single mean atlas, and, progressively, evolved to multi-atlas strategies where decision fusion strategies are involved [10,25,26] together with label propagation. Classifier fusion, based on the majority vote rule, has been shown to be accurate for segmenting brain structures. This strategy can become more robust and increasingly accurate as the number of classifiers grows. However, it suffers from problems of scale when the number of atlases is large. [26] compares different classifier selection strategies, which are applied to a group of 275 subjects with manually labeled brain MRI. An adaptive multi-atlas segmentation method (AMAS) is presented in [10]. AMAS includes an automated decision to select the most appropriate atlases for a target image and a stopping criterion for registering atlases when no further improvement is expected. This method obtained the best mark in the CAUSE07 challenge.

b) Different ways of exploiting supervised learning in segmentation methods have been incorporated. In [27], the atlas-based segmentation method presented uses segmentation confidence maps, which are learned from a small manually-segmented training set, and incorporated into the cost term. This cost is responsible for weighting the influence of initial segmentations in the multi-structure registration. Moreover, multiple atlases are used both in a supervised atlas-correction step, and multiple atlas propagation. In [28], a two-stage method is presented, which benefits from capabilities of mathematical feature extractors and artificial neural networks. In the first stage, geometric moment invariants (GMIs) are applied at different scales to extract features that represent the shapes of the structures. Next, multi-dimensional feature vectors are constructed that contain the GMIs along with image intensity values, probability atlas values (PAVs), and voxel coordinates. These feature vectors are used to estimate signed distance maps (SDMs) of the desired structures. To this end, multi-layer perceptron neural networks (MLP-NN) are designed to approximate the SDMs of the target structures. In the second stage, the estimated SDM of each structure is used to design another MLP-NN to classify the image voxels into two classes: inside and outside the structure.

c) Shape and appearance models involve establishing correspondence across a training set and learning the statistics of shape and intensity variation using PCA models. To segment an image being studied, model parameters which best approximate the structures have to be computed. [29] applies an active appearance model (AAM)-based method to segment the caudate nucleus. A "composite" 3D profile AAM is constructed from the surfaces of several subcortical structures using a training set, and individual AAMs of the left and right caudate are constructed from a different training set. Segmentation starts with affine registration to initialize the composite model within the image. Then, a search is performed using the composite model. This provides a reliable but coarse segmentation, used to initialize a search with the individual caudate models. [30] uses a statistical shape model with elastic deformations to segment the hippocampus, thalamus, putamen, and pallidum. In [31], a comparison of four different strategies of brain subcortical structure segmentation is presented: two of them are atlas-based strategies ( [26] and [17]) and the other two are based on statistical models of shape and appearance ( [29] and [32]). The best results are achieved by the multi-atlas classifier fusion and labeling approach [26] which treats atlases as classifiers and combines them using a majority voting rule.

d) With reference to energy-minimization methods, [33] uses a deformable mesh followed by normalized cuts criterion to segment the caudate and the putamen from PET images. [34] proposes a multiphase level set framework for image segmentation using the Mumford-Shah model, as a generalization of an active contour model. In [35], a method is presented for the segmentation of anatomical structures, which incorporates prior information about the intensity and curvature profile of the structure from a training set of images and boundaries. In [36], the GC strategy is adapted for segmenting anatomical brain regions of interest in diffusion tensor MRI (DT-MRI). An open source application called ITK-SNAP was developed [37] for level set segmentation.

Finally, there exist some libraries, such as Freesurfer [38], Slicer [39], and SPM [21], which have been developed to address the MRI segmentation problem. However, all of them are limited to atlas-based algorithms which lack robustness when dealing with different types of subjects. Hence, constructing an hybrid approach that combines atlas-based and energy-based strategies is a natural extension of state-of-the-art algorithms. The combination presented in this paper exploits atlas structure information and an adaptive ad hoc energy model. Moreover, the proposed energy model also takes advantage of supervised learning techniques.

## 3 CaudateCut

In this section, we review the GC framework and describe the novel CaudateCut segmentation algorithm. Table 1 summarizes the terminology used in the next sections.

**Table 1:** Table of terms

Ω(*L*_*p*_,*L*_*q*_)	Pulse function.
*δ*	Trade off coefficient between *U *and *B*.
∂_*k*_	Substraction of graylevel for a pair of bins in *C*_*c*×*r*_.
$\frac{\partial }{\partial x}\frac{\partial^{2}}{\partial x^{2}}$	First and second derivatives w.r.t. *x*.
*θ*_{*p*,*q*}_	Angle between minimum gradient variation vectors in pixel *p *and *q*.
*α*, *σ *and *β*	Weight parameters of boundary term.
|·|	Cardinal of a set.
C,B	Sets of caudate and background seeds.
*C*_*c*×*r*_	Correlogram structure of *c *circles and *r *radius.
**d***p*	Correlogram descriptor for pixel *p*.
*d*(·,·)	Minimum Euclidean distance between two sets of voxels.
$\text{Dilat}{\text{e}}_{k_{d}}\left(.\right)$	Dilatation function of structure element of *k*_*d *_pixels.
*E*(·),*U*(·),*B*(·)	Cost function, Unary term, and Boundary term.
$\text{Erod}{\text{e}}_{k_{e}}\left(.\right)$	Erosion function of structure element of *k*_*e *_pixels.
G=<V,E>	Graph of nodes V and edges *ε *.
*G *and ℓ	The 2-dimensional Gaussian function and the Lindeberg parameter.
*H*(·)	Entropy value.
*I *and *I*_*p*_	Grayscale image and image intensity value at pixel *p.*
*J*_*p*,*γ*,*s*_	Binary edge map of pixel *p *at scale *s *and with sensitivity edge threshold *γ*.
$L=\left(L_{1},...,L_{p},...,L_{\left|P\right|}\right)$	Binary vectors of assignments to pixels p∈P.
N	Set of unordered pairs {*p*, *q*} of neighboring pixels under a 4-(8-) neighborhood system.
*N*_{*p*,*q*} _, *O*_{*p*,*q*}_	Boundary terms based on first and second derivatives.
$P=\left(1,...,p,...,|P|\right)$	Set of indexes of *I*.
P_u_(·), P_s_(·)	Unsupervised and supervised probability function.
P(·)	General frequency-based Probability function.
{*p*,*q*}	*n*-link connecting a pair of neighbors *p *and *q*.
$R_{0}$	Region of Interest (ROI).
*R*_*p*_(*s*)	Neighborhood of size *s *× *s*.
*S*, *T*	Caudate and Background terminal nodes.
SVM(.)	Support Vector Machines classifier function.
*T*_*p*_	Atlas-based threshold over probability map.
*UU*(·),*SU*(·)	Unsupervised and supervised unary terms.
$X=\left({\text{x}}_{1},...,{\text{x}}_{p},...,{\text{x}}_{\left|P\right|}\right)$	Set of pixels of *I*.

### 3.1 Graph-Cut Framework

In this section, we introduce the GC framework used in the CaudateCut segmentation algorithm. Let us define $X=\left({\text{x}}_{1},...,{\text{x}}_{p},...,{\text{x}}_{\left|P\right|}\right)$ as the set of pixels for a given grayscale image *I*; $P=\left(1,...,p,...,|P|\right)$ as the set of indexes for *I*; N as the set of unordered pairs {*p*, *q*} of neighboring pixels of P under a 4-(8-) neighborhood system, and $L=\left(L_{1},...,L_{p},...,L_{\left|P\right|}\right)$ as a binary vector whose components *L*_*p *_specify assignments to pixels p∈P. Each *L*_*p *_can be either "foreground" or "background", or equivalently "cau" or "back" for our problem (abbreviations for caudate and background), indicating whether pixel *p *belongs to the caudate or background, respectively. Thus, the array *L *defines a segmentation of image *I*. The GC formulation defines the cost function *E*(*L*) which describes soft constraints imposed on boundary and region properties of *L*:

(1) E(L)=U(L)+δB(L),

where *U*(*L*) is the unary term (or region properties term),

$$U\left(L\right)=\underset{p\in P}{\sum }U_{p}\left(L_{p}\right),$$

and *B*(*L*) is the boundary property term,

$$B\left(L\right)=\underset{\left\{p,q\right\}\in N}{\sum }B_{\left\{p,q\right\}}\Omega \left(L_{p},L_{q}\right),$$

where,

$$\Omega \left(L_{p},L_{q}\right)=\left\{\begin{array}{cc} 0, & \text{if}\;L_{p}\neq L_{q}\\ 1, & \text{otherwise}. \end{array}\right)$$

The coefficient *δ *∈ ℝ^+ ^in Eq.(1) specifies the relative importance of the unary term *U*(*L*) compared to the boundary term *B*(*L*). The unary term *U*(*L*) assumes that the individual penalties for assigning pixel *p *to "cau" and "back", correspondingly *U*_*p*_("cau") and *U*_*p*_("back"), are given. The term *B*(*L*) comprises the boundary properties of segmentation *L*. Coefficients *B*_{*p*,*q*} _≥ 0 should be interpreted as a penalty for a discontinuity between *p *and *q.*

The GC method imposes hard constraints on the segmentation results by means of the definition of seed points where labels are predefined and cannot be modified. The subsets C⊂P,B⊂P,C∩B=∅ denote the subsets of caudate and background seeds, respectively. The goal of GC is to compute the global minimum of Eq. (1) from all segmentations *L *satisfying the hard constraints ∀p∈C, *L*_*p *_= "cau", ∀p∈B, *L*_*p *_= "back".

Let us describe the details of the graph created to segment an MRI image. A graph G=<V,E> is created with nodes, V, corresponding to pixels p∈P of the image plus two additional nodes: the caudate terminal (a source *S*) and the background terminal (a sink *T*), therefore, V=P∪{S,T}. The set of edges *ε *consists of two types of undirected edges: *n*-links (neighborhood links) and *t *-links (terminal links). Each pixel *p *has two *t*-links {*p*, *S*} and {*p*, *T*} connecting it to each terminal. Each pair of neighboring pixels {*p*, *q*} in N is connected by an *n*-link. Without introducing any ambiguity, an *n*-link connecting a pair of neighbors *p *and *q *will be denoted by {*p*, *q*}, giving $E=N{⋃}_{p\in P}\left\{\left\{p,S\right\},\left\{p,T\right\}\right\}$. Final segmentation is then computed over the defined graph using the min-cut algorithm to minimize *E*(*L*).

### 3.2 CaudateCut Segmentation Algorithm

In this section, we describe the steps in the automatic caudate segmentation algorithm in detail. The CaudateCut algorithm is summarized in Table 2.

**Table 2:** Automatic CaudateCut Segmentation Algorithm

1.	Initial segmentation using AB method.
2.	Set background and caudate seeds by erosion and dilatation of AB mask.
3.	Initialize unsupervised unary potentials *UU*_*p*_("*cau*") and *UU*_*p*_("*back*") based on local graylevel intensities.
4.	Initialize supervised unary potentials *SU*_*p*_("*cau*") and *SU*_*p*_("*back*") based on SVM correlogram classifier.
5.	Initialize unary term based on combined unary potentials.
6.	Initialize boundary term *B*(*L*) based on first and second derivatives of intensities and multi-scale edge map.
7.	Estimate caudate segmentation using GC.

#### 3.2.1 Atlas-based Segmentation

**Figure 2:**
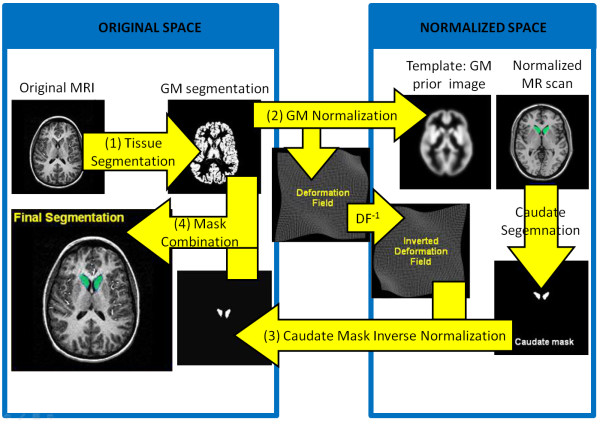
**Flowchart of the atlas-based segmentation approach**. Flowchart of the atlas-based segmentation approach used in this work.

In this work, the atlas-based segmentation of the caudate largely follows the strategy proposed by [18]. The main steps in the algorithm are illustrated in Figure 2 and described thus:

1. First, a non-uniformity image intensity correction is computed. Then, the corrected image is classified into WM, GM, and CSF.

2. In the next step, the GM image is elastically registered from its original geometrical space to match a template image (which represents the expected distribution of gray matter in the subjects under study) in the so-called normalized space. The deformation field obtained is inverted to map the normalized space onto the original space.

3. This inverted deformation is applied to the caudate segmentation in the normalized space, thus yielding a first segmentation of the caudate nucleus of the subject.

4. Finally, in order to refine this first segmentation, the GM mask of the subject under study is combined with the mask obtained by unwarping the normalized caudate segmentation. They are combined as follows: the GM and caudate probability maps are multiplied and a threshold *T*_*p *_is imposed over the result: we consider that a voxel belongs to the caudate only where the product map is larger than *T*_*p*_.

**Figure 3:**
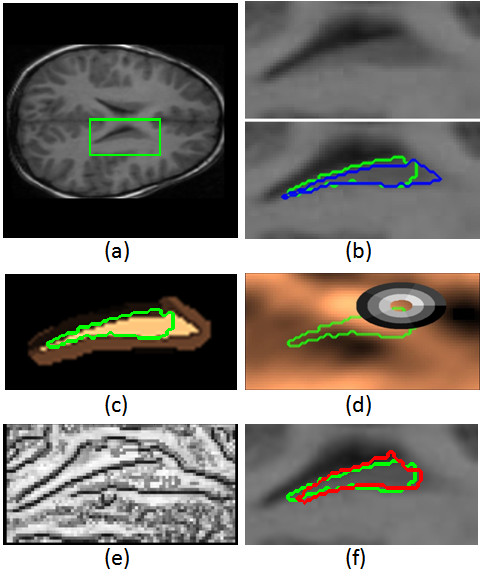
**Framework steps**. (a) Original MRI scan, (b) Top: Crop from the MRI scan, Bottom: Atlas-based segmentation (blue) and GT (green), (c) Unsupervised probability values P_u_(*L*_*p *_= "cau") and GT (green), (d) Supervised probability values P_s_(*L*_*p *_= "cau") and GT (green), (e) Boundary potentials *B*(*L*), (f) Image crop, GT (green) and CaudateCut result (red).

This atlas-based segmentation method depends strongly on the atlas definition. In some situations, this can result in a solution that does not fit the target structure well and a further refinement may be necessary. However, the segmentation obtained may be useful for roughly locating the region of interest, and thus, it can be used to define the seeds for GC application. Figure 3 (b) shows the result of AB segmentation for the input image in Figure 3 (a).

### 3.2.2 Seed Initialization

GC is a semi-automatic interactive method, since the seeds are manually defined. In order to achieve a fully automatic method, we use the result of the atlas-based method to define an initial segmentation taking advantage of the atlas caudate shape. We define caudate and background seeds by performing morphological operations on the ROI obtained $R_{0}$ in the atlas-based mask. To define the caudate seeds, C, we compute $C=\text{Erod}{\text{e}}_{k_{e}}\left(R_{0}\right)$, where $\text{Erod}{\text{e}}_{k_{e}}$ denotes an erosion with a structural element of *k*_*e *_pixels. In the case of background seeds, we dilate the region $R_{0}$ and keep the complementary set, $B=P\backslash \text{Dilat}{\text{e}}_{k_{d}}\left(R_{0}\right)$, where $\text{Dilat}{\text{e}}_{k_{d}}$ denotes a dilatation with a structural element of *k*_*d *_pixels. In the example shown in Figure 3 (a), the selection of C and B seeds is obtained from erosion and dilation of the AB segmentation shown in Figure 3 (b).

#### 3.2.3 Unary Energy Term

In this section, we describe how to compute the unary energy term for the GC energy function. This energy term is divided into two: an unsupervised part and a supervised part. The unsupervised part is computed in a problem-dependent image way, based on the graylevel distribution of the seed pixels. The supervised part is computed from a support vector machine classifier (SVM) based on the contextual learning of caudate derivatives. Next, we describe in detail both parts of the unary term and the final combination.

##### Unsupervised unary term

We define the unsupervised unary term using caudate and background models based on graylevel information pertaining to the seeds. We initialize the unary potentials at each pixel *p *as,

$$\begin{array}{ll} UU_{p}\left(\text{”cau”}\right) & =-\ln\left({\text{P}}_{u}\left(L_{p}=\text{”cau”}\right)\right),\;\\ UU_{p}\left(\text{”back”}\right) & =-\ln\left({\text{P}}_{u}\left(L_{p}=\text{”back”}\right)\right).\;\\ & \; \end{array}$$

The probability of a pixel *p *being marked as "cau", P_*u*_(*L*_*p *_= "cau"), is computed using the histogram of graylevels of caudate seeds. The probability of a pixel being marked as "back" is computed using the inverse probabil-ity, as P_u_(*L*_*p *_= "back") = 1-P_*u*_(*L*_*p *_= "cau"), since background seeds contain GM, WM and CSF and it is difficult to extract a model directly from them. Figure 3 (c) shows the unsupervised probability values P_*u*_(*L*_*p *_= "cau")) for the image in Figure 3 (a).

The unsupervised unary term estimates image-dependent caudate pixel probabilities based on caudate seeds. However, given the noisy information of MRI images and the small number of caudate seed pixels, a high generalization based on this term is not always guaranteed. In this context, we propose using a combination of the unsupervised energy with the supervised, which is based on learning contextual caudate derivatives from Ground Truth (GT) data.

##### Supervised unary term

In order to define the supervised unary term, we train a binary classifier using a set of MRI slices as a training set. In particular, we extract a pixel descriptor using a correlogram structure. The correlogram structure captures contextual intensity relations from circular bins around the pixel analyzed [40].

Given a pixel *p*, a correlogram *C*_*c*×*r *_is defined, where *c *and *r *define the number of circles and radius of the structure. Then, each bin *b *from the set of *n *bins, with *n *= *c *⋅ *r*, is defined as the area delimited by two consecutive circles of the given radius. Given the pixel *p *and its correlogram structure $C_{c\times r}^{p}$, its supervised caudate descriptor is defined as:

$$d_{p}=\left\{\partial_{1},..,\partial_{k},..,\partial_{n\cdot \left(n-1\right)∕2}\right\},$$

where ∂_*k *_is the signed substraction of graylevel information within a pair of bins in *C*_*c*×*r*_. In this sense, the descriptor contains the *n *· (*n *- 1)/2 graylevel derivatives of all pairs of bins within *C*_*c*×*r*_, which captures all spatial relations of graylevel intensities in the neighborhood of *p*. An example of a correlogram structure estimated for a caudate pixel is shown in Figure 3 (d).

We extract the descriptors for a subset of pixels on C and B from the training set data. Given the set of descriptors, a linear SVM classifier is trained in order to predict caudate confidence on image pixels from new test data. In our case, we use the output confidence of the classifier as a measure of the "probability" of a pixel belonging to the caudate. Then, the supervised unary potentials at each pixel *p *are:

SUp(''cau'')=−ln(Ps(Lp=''cau'')),SUp(''back'')=−ln(Ps(Lp=''back'')).

The probability of a pixel being marked as "cau" is computed using the confidence of the SVM classifier over its correlogram descriptor P_*s*_(*L*_*p *_= "cau") = SVM(*p*). The probability of a pixel being marked as "back" is computed as the negative of the output margin of the classifier P_*s*_(*L*_*p *_= "back") = -SVM(*p*). Figure 3 (d) shows the supervised caudate probability values P_*s*_(*L*_*p *_= "cau")) for the image in Figure 3 (b).

##### Combined unary term

The final unary term is defined as the addition of the unsupervised and supervised values at pixel *p *as follows:

Up(''cau'')=UUp(''cau'')+SUp(''cau''),Up(''back'')=UUp(''back'')+SUp(''back''),

#### 3.2.4 Boundary Energy Term

To define boundary potentials, we use first and second intensity derivatives of the image to use the intensity and geometric information. Moreover, given the high variability in contrast between the caudate and background in different parts of the images, we propose weighting the boundary term using an image-dependent multi-scale edgeness measure.

Specifically, we define the boundary potentials as the following convex linear combination:

$$B_{\left\{p,q\right\}}=j\left(\alpha N_{\left\{p,q\right\}}+\left(1-\alpha \right)O_{\left\{p,q\right\}}\right).$$

First, we define *N*_{*p*, *q*} _and *O*_{*p*, *q*} _as:

(2) N{p,q}=1xp-xq2 exp-(Ip-Iq)22σ2,O{p,q}=1xp-xq2 exp-θ{p,q}22β2.

The term *θ*_{*p*,*q*} _denotes the angle between two unitary vectors codifying the directions of minimum gradient variation in pixel *p *and *q *based on the Hessian eigenvectors. In particular, we choose the direction of the eigenvector of the Hessian matrix with the smallest eigenvalue which gives the direction of the smallest variation at each pixel. The parameter *α *is empirically set by cross-validation, while *σ *and *β *are computed by adapting the image distribution to *I*_*p *_and *θ*_{*p*,*q*}_, respectively. Intuitively, the function *N*_{*p*, *q*} _penalizes discontinuities between pixels of similar intensities and *O*_{*p*,*q*} _penalizes for discontinuities between pixels of similar gradient variations.

The differential operators involved in the previous definition (Eq. 2) are well-posed concepts of linear scale-space theory, defined as convolutions with derivatives of Gaussians: $\frac{\partial }{\partial x}I\left(x,s\right)=s^{\ell }I\left(x\right)*\frac{\partial }{\partial x}G\left(x,s\right)$, where *G *is the 2-dimensional Gaussian function and ℓ is the Lindeberg parameter.

The selection of the Gaussian scale parameter is crucial for obtaining a satisfactory result. For a given pixel *p*, we consider an *s *× *s *neighborhood *R*_*p*_(*s*), and measure its entropy value:

$$H\left(R_{p}\left(s\right)\right)=-\overset{r}{\underset{i=1}{\sum }}P\left(i|R_{p}\left(s\right)\right)\log_{2}\text{P}\left(i|R_{p}\left(s\right)\right),$$

where P(*i*|*R*_*p*_(*s*)) is the probability of taking the value *i *in the local region *R*_*p*_(*s*), with *r *being all the possible discrete values. The scale chosen is defined by the maxima of the function *H *in the space of scales $S_{p}=\left\{s:\partial H\left(R_{p}\left(s\right)\right)∕\partial s=0,\partial^{2}H\left(R_{p}\left(s\right)\right)∕\partial s^{2}<0\right\}$.

Second, we define the *J *term as the multi-scale edgeness measure at each pixel: $J=\left(J_{1}^{*},...,J_{p}^{*},...,J_{\left|P\right|}^{*}\right)$. In order to compute $J_{p}^{*}$, we first run the Canny edge detector algorithm on the observed image at different threshold levels. Then, we compute the edge probability at each pixel by linear averaging of the edge thresholds for different scales as follows:

$$J_{p}^{*}=\underset{j}{\min}\frac{1}{n}\overset{n}{\underset{k=1}{\sum }}J_{p,\gamma k,s_{j}},$$

where *J*_*p*,*γk*,*sj *_is the binary edge map using threshold *γ*_*k *_and scale *s*_*j *_for pixel *p*. If pixel *p *is labeled as an edge pixel for most of the threshold levels at a significant scale, it has a high probability of being an edge pixel. In order to decrease the smoothness effect at the regions near a boundary, we convolve the probability map with a Gaussian kernel. Figure 3 (e) shows the boundary potential values *B*(*L*) for the image in Figure 3 (a). Intuitively, the term *J *adaptively changes the influence of the boundary term for pixels in the image, since boundary regions should be less regularized than the rest of the image regions.

Finally, by applying the min-cut algorithm over the defined energy function and image graph, we obtain the final caudate segmentation. Figure 3 (f) shows the segmentation resulting from applying the CaudateCut algorithm.

## 4 Experimental Section

Before presenting our results, we first describe the material and methods of comparison, and also the validation protocol for the experiments.

### 4.1 Material

We considered two different databases, named URNC database and IBSR database, in order to validate the CaudateCut method we have proposed.

• **URNC database**. This is a new database, which includes 39 children (35 boys and 4 girls) with ADHD, according to DSM-IV, referred from the Unit of Child Psychiatry at the Vall d [001]ebron Hospital in Barcelona, Spain, and coordinated by the Unit of Research in Cognitive Neuroscience (URNC) at the IMIM Foundation, together with 39 control subjects (27 boys and 12 girls) recruited from the community. The mean age of the groups was 10.8 (S.D.: 2.9) and 11.7 (S.D.: 3), respectively. The groups were matched for handedness and IQ. The 1.5-T system was used to acquire brain MRI scans. The resolution of the scans is 256 × 256 × 60 pixels with 2-mm thick slices. Expert segmentations of the 79 individual caudate nuclei was obtained. MRIcro software^1 ^was used for volume labeling and manipulation.

• **IBSR database**. This dataset is part of a public database released by CAUSE07 Challenge [9]. It is composed by 18 T1-weighted MRI scans from the Internet Brain Segmentation Repository (IBSR). It also contains expert segmentations of caudate structure. The MRI scans are of 1.5 mm thickness. Originally, the data size was 256 × 128 × 256 pixels, but in order to prepare data for the later application of the CaudateCut algorithm, we re-oriented they data by *X*-axis rotation and converted it into 256 × 256 × 128 pixels. For more details of the acquisition, visit CAUSE07 Challenge website^2 ^from were the data was downloaded.

**Figure 4:**
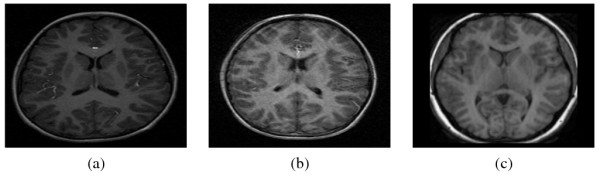
**Sample control and ADHD MRIs from URNC and IBSR databases**. Control (a) and ADHD (b) example MRI slices from the URNC database and an example MRI slice from the IBSR database (c).

Figure 4 displays a sample control (a) and ADHD (b) MRI from the URNC database and a sample MRI from the IBSR database, (c). As can be appreciated, the quality of the ADHD image is worse than that of the control image, probably due to the movement of the children during image acquisition. Anisotropic filtering [41] was performed on all the slices before CaudateCut was applied.

### 4.2 Methods

We compared the CaudateCut method to two state-of-the-art methods: a classical atlas-based method, and a multi-atlas segmentation method. We also compared the results with the inter-observer (IO) variability of the expert GT.

#### AB method

We implemented atlas-based segmentation of the caudate following the strategy presented in [18]. To this end we used the SPM toolbox implementation of the unified non-linear normalization and tissue segmentation. The parameters of the method were set by default as in the SPM8 implementation, except for the threshold *T*_*p*_, which was estimated using a subset of 5 control subjects from the URNC database and set to *T*_*p *_= 0.1. The method was implemented using Matlab2008.

#### AMAS method

An adaptive multi-atlas segmentation method (AMAS) was implemented as presented in [10]. For the atlas selection strategy we computed the absolute voxelwise difference between the target image and the registered images from the atlases and ordered them from smallest to largest. Then, the atlas information was propagated until the stopping criterion was reached. The stopping criterion was defined by the percentage of voxels that change their segmentation label after a new atlas propagation. This threshold was set to 0.05 for all experiments. The rest of the parameters in the AMAS method were set as described in [10]. The method was implemented using Matlab2008, and elastix6 version 3.9^3 ^was used for volume registration, as suggested in [10].

#### CaudateCut method

The CaudateCut method was implemented using Matlab2008 and the SPM toolbox. In all the experiments the parameters were set to *k*_*e *_= 4, *k*_*d *_= 10, *c *= 3, *r *= 5, *α *= 0.5, $S_{p}=\left[1,1.5,...,6\right]$, ℓ = 0, *γ*_*k *_∈ [0.02,0.03,..., 0.3] and *s*_*j *_∈ [0.5,1,..., 5]. The parameters *σ *and *β *were estimated for each image, as explained above. The parameter *δ *was tuned by cross-validation and was set to 50 for the URNC dataset and 100 for the IBSR database. In order to train the SVM classifiers for computation of the supervised unary term, we performed a subsampling of pixels from each slice. In particular, we took all the pixels labeled as caudate in the GT, and the same number of background pixels. The background pixels were subsampled in a stratified way, trying to select pixels from all parts of the background.

#### Manual method

Experts use MRIcro [42] to manually delineate the caudate boundaries slice by slice. See [1] for more details of the procedure.

### 4.3 Validation

The quality of a segmentation can be evaluated in many different ways. Plausible evaluation criteria may depend on the purpose of the segmentation procedure. In order to be sufficiently general, we evaluated several volumetric measures, as well as voxel by voxel comparison measures. We focused on the six metrics detailed below, as proposed in [9]. In all of them, R corresponds to the estimated segmentation, G to the GT segmentation and | · | denotes the cardinal of a set.

1. Volumetric similarity index (or mean overlap), in percent:

$$\text{SI}=2\left|\frac{\text{R}\cap \text{G}}{R+G}\right|\cdot 100.$$

2. Volumetric union overlap, in percent:

$$\text{VO}=\left|\frac{\text{R}\cap \text{G}}{\text{R}\cup \text{G}}\right|\cdot 100.$$

3. Relative absolute volume difference, in percent:

$$\text{VD}=\left|\frac{\text{VO}{\text{L}}_{\text{R}}-\text{VO}{\text{L}}_{\text{G}}}{\text{VO}{\text{L}}_{\text{G}}}\right|\cdot 100,$$

     where VOL_R _and VOL_G _correspond to the total volume of the *R *and *G *segmentations, respectively.

4. Average symmetric surface distance, in millimeters:

$$\text{AD}=\frac{\left(\overset{N}{\underset{i=1}{\sum }}\text{d}{\left({\text{B}}_{\text{S}i},{\text{B}}_{\text{R}}\right)}^{2}+\overset{M}{\underset{i=1}{\sum }}\text{d}{\left({\text{B}}_{\text{S}},{\text{B}}_{\text{R}i}\right)}^{2}\right)}{\left|B_{S}\right|\cdot \left|B_{R}\right|},$$

     where *B*_*S *_and *B*_*R *_correspond to the set of border voxels in R and G, respectively, and *d*(·,·) returns the minimum Euclidean distance between two sets of voxels.

5. Root Mean Square (RMS) symmetric surface distance, in millimeters:

$$\text{RMSD}=\sqrt{AD}.$$

6. Maximum symmetric surface distance, in millimeters:

$$\text{MD}=\underset{i,j}{\max}\left(\text{d}\left({\text{B}}_{\text{S}i},{\text{B}}_{\text{R}}\right),\text{d}\left({\text{B}}_{\text{S}i},{\text{B}}_{\text{R}j}\right)\right).$$

Note that the volumetric measures VO and SI have 100 as a perfect segmentation and 0 as the lowest possible value, when there is no overlap at all between the estimated segmentation and GT. In the case of VD, the perfect value is 0, which can also be obtained for a non-perfect segmentation, as long as the volume of that segmentation is equal to the volume of the reference. For voxel comparison measures, AD, RMSD and MD, the perfect value is 0.

In order to validate the AMAS and CaudateCut methods (SVM classifiers for supervised unary term computation), we followed a leave-one-out strategy. Finally, Student's paired t-test [43] was used to evaluate the statistical significance between pairs of segmentation algorithms with a particular dataset (threshold of *p *< 0.05). The null hypothesis corresponds to the hypothesis that the two groups belong to the same distribution and is called *H*_0_. Matlab2008 was used to perform this test.

### 4.4 Results and Discussion

We divide the results into two sections corresponding to two related experiments: segmentation evaluation and ADHD volumetric quantitative analysis.

#### 4.4.1 Segmentation Evaluation

##### A) Quantitative segmentation results

We compared the performance of the CaudateCut, AMAS and AB methods. Table 3 shows the results obtained in the experiments on both URNC and IBSR datasets. For all six validation measures, our proposed CaudateCut produced better results than both AB and AMAS for both databases. With regard to the volumetric measures, CaudateCut achieved good mean rates of 80, 75% for SI, 68, 02% for VO, and 16, 22 for VD. Voxel by voxel mean measures are also acceptable, with 0.0024 mm for AD, 0.0733 mm for RMSD, and 35.70 mm for MD. The large MD values are due to the recurrent errors present in the internal boundaries of the caudate defined between caudate head and body, as is clarified in the visual results below. For the IBSR database, the AMAS method obtained larger VO and SI values than the AB method, whereas, in the URNC database, the AB method improved on the result of the AMAS method. This could be due to the fact that the AB parameters were tuned in the URNC database. In this sense, CaudateCut was able to properly overcome this inconvenience and improve on the AMAS results in the IBSR database. It is important to note that CaudateCut showed robustness to AB performance.

**Table 3:** Quantitative results of AB, AMAS and CaudateCut

Database	Method	SI	VO	VD	AD	RMSD	MD
	AB	79.85	66.55	9.63	0.0029	0.0861	48.31
URNC	AMAS	66.67	51.45	24.81	0.0091	0.0913	48.11
	CaudateCut	**82.60**	**70.49**	**9.10**	**0.0028**	**0.0780**	**47.97**

	AB	74.02	58.85	23.34	0.0030	0.0950	30.86
IBSR	AMAS	75.00	60.14	25.54	0.0024	0.0750	28.37
	CaudateCut	**78.91**	**65.55**	**17.80**	**0.0019**	**0.0687**	**23.43**

Quantitative results of AB, AMAS and CaudateCut methods applied to URNC and IBSR databases. Validation measures are, SI: volumetric similarity index (in %); VO: volumetric overlap (in %); VD: relative absolute volume difference (in %); AD: average symmetric surface distance (in mm); RMSD: root mean square symmetric surface distance (in mm); MD: maximum symmetric surface distance (in mm).

##### B) Qualitative segmentation results

**Figure 5:**
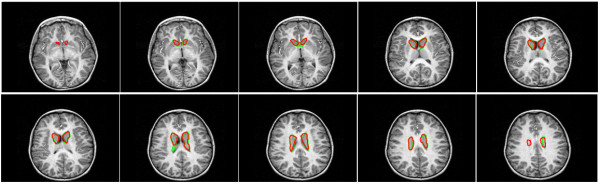
**Qualitative CaudateCut results**. Example of CaudateCut results. GT is shown in green and CaudateCut segmentation in red.

Figure 5 shows qualitative CaudateCut results for the MRI slices of a control subject. In most of the slices, the CaudateCut segmentation result (red line) is highly comparable to the GT (green line). However, segmentation differences occur in the first and last caudate frames, where some voxels are classified as caudate by CaudateCut, but not by the GT (false positives). The inherent ambiguity of the caudate boundaries makes the expert's task of manually defining the caudate start and end slices arduous. This introduces variability and produces errors in MRI atlas information corresponding to the end slices. It is difficult for CaudateCutThis to rectify this kind of error. The AB method introduces fake seeds in these positions and CaudateCut propagates these errors, since it can not remove the seeds. In the second column of the second row, some voxels are not classified as caudate, while they should be, according to GT (false negatives). This particular sample slice corresponds to the transition between caudate head and body, where the caudate shape changes abruptly from the rounded head to the elongated body [5]. Due to the intra-subject variability of the caudate shape, in the caudate internal transition, atlas priors are less reliable and introduce errors. This inconvenience, together with the lack of a well-contrasted boundary defining the caudate body in the first slices, makes these mistakes difficult to be rectify.

**Figure 6:**
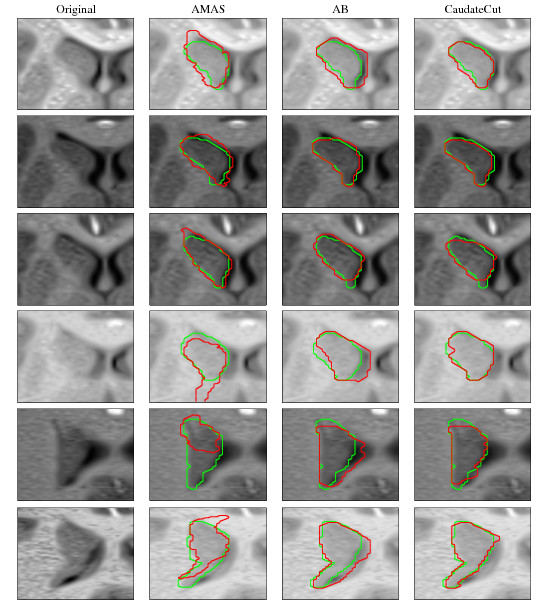
**Segmentation comparison of AMAS, AB, and CaudateCut**. Some left caudate segmentation results from the URNC database. First column: Original image crop. Second column: AMAS result. Third column: AB result. Fourth column: CaudateCut result. GT is shown in green and automatic segmentation result in red.

Figure 6 compares qualitative results of left caudate segmentation of URNC database MRI slices using the AMAS (second column), AB (third column) and CaudateCut (fourth column) methods. Note that the best segmentation results were obtained by the novel CaudateCut segmentation method, followed by AB, and finally, by the AMAS strategy. In general, CaudateCut improves AB segmentation and obtains a better fit to the caudate boundaries. Only in a few cases (examples in rows 2 and 3), does CaudateCut agree with the AB segmentation, and the GC strategy did not apply changes to the final segmentation. It can be seen that the registration strategy applied for the AMAS method was unable to correctly fit the caudate boundaries. At several locations, the caudate boundaries are not clearly defined. For example, in the first row example, the lower boundary mostly consists of partial volume effect voxels. Thus, the caudate was over-segmented by all the methods.

##### C) Statistical analysis

We performed four different statistical t-tests: CaudateCut vs. AB and CaudateCut vs. AMAS for the two databases (URNC and IBSR) based on the VO measure. Table 4 presents the results of the tests. In the table, the t-test result is true (accept *H*_0_) or false (reject *H*_0_), *t *is Student's t statistic, *p *represents the p-value, and CI means the confidence interval of differences. The results of the four tests were favorable for CaudateCut, showing that the differences in the overlap measures between CaudateCut and AB and AMAS were significant.

**Table 4:** Statistical test results

Database	Test	t-test	*t*	*p*	CI(95%)
	CaudateCut vs. AB	**false**	4.08	0.0001	0.02 to 0.0586
URNC	CaudateCut vs. AMAS	**false**	11.36	3.28-10^-18^	0.24 to 0.34

IBSR	CaudateCut vs. AB	**false**	3.23	0.0028	0.0248 to 0.1092
	CaudateCut vs. AMAS	**false**	2.49	0.0177	0.01 to 0.0982

Four different statistical t-tests for the two databases: URNC (first row) and IBSR (second row). In the third to sixth columns: t-test, t-test result; *t*, Student's t statistic; *p*, p-value; CI, confidence interval of differences.

##### D) Difference analysis

**Figure 7:**
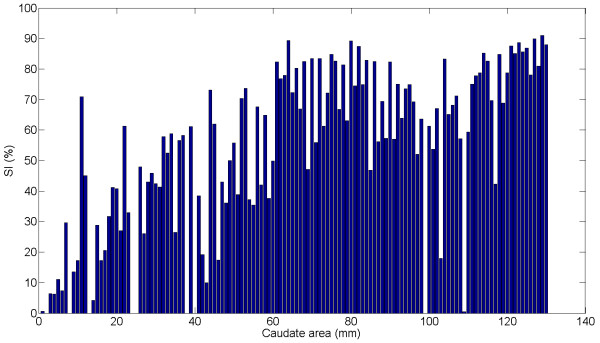
**SI values using CaudateCut **Similarity index (SI) values (in %) depending on the caudate area (in mm) for the slices.

Figure 7 shows the values of SI obtained using CaudateCut depending on the area of the slice of the caudate nucleus. As can be seen, the SI values are lower for smaller areas and tend to increase for larger caudate regions. This corroborates the claim that smaller structures are more difficult to automatically segment.

##### E) Computational differences

Concerning the computational time, AMAS was the most costly method in terms of testing time, since multiple registration had to be performed for each subject segmentation. On average, 2-3 registrations were performed for each volume segmentation and each registration took 7 minutes on a standard high-end PC, thus making 17.5 minutes for the whole volume segmentation. The AB method was the fastest, taking around 5 minutes on average for the whole volume segmentation. CaudateCut involves applying the AB method and later the GC minimization process method. The total time was around 6 minutes for the whole volume segmentation.

##### F) Inter-observer variability

Finally, the inter-observer variability was computed using manual left caudate segmentations from the URNC database by means of two different experts. Table 5 shows the validation measures computed using these two GT segmentations. As mentioned in the introduction, obtaining an accurate manual segmentation is difficult even for experts, because of the low contrast and resolution of the caudate regions. Note that the measure values are comparable to those obtained with CaudateCut.

**Table 5:** Quantitative Measures of IO Variability

Database	SI	VO	VD	AD	RMSD	MD
IO on URNC	80.56	67.80	22.84	0.003	0.092	92.54

Quantitative measures of IO variability for URNC databases. Validation measures are, SI: volumetric similarity index (in %); VO: volumetric overlap (in %); VD: relative absolute volume difference (in %); AD: average symmetric surface distance (in mm); RMSD: root mean square symmetric surface distance (in mm); MD: maximum symmetric surface distance (in mm).

#### 4.4.2 ADHD Volumetric Quantitative Analysis

The a priori hypothesis that developmental anomalies exist in the caudate nucleus of people with ADHD is generally accepted. Previous imaging studies have analyzed this hypothesis [4,2,3].

In this work, we analyzed right and left caudate volumetric differences between ADHD and control subjects in the URNC database. To this end, we performed a comparison of mean volume values applying Student's t-test for independent samples (with a threshold of *p *< 0.05). The aim of this experiment was to show that the analysis performed using automatic CaudateCut segmentation was coherent with the results of manual analysis. To carry out the manual and automated statistical analysis we considered GT and CaudateCut segmentations, respectively. ROI measures in voxels were transformed into cubic millimeters, mm^3 ^(ROI total number of voxels multiplied by voxel dimensions).

Table 6 and Table 7 show the results of the manual and automatic analyses, respectively. Both tables contain mean volume measures, and standard deviation of control and ADHD groups for the right and left caudate separately. Moreover, the results of Student's t-tests are presented: the t-test corresponds to a true (accept *H*_0_) or false (reject *H*_0_) result, *t *is Student's t statistic, *p *represents the p-value, and CI means the confidence interval of differences. As can be observed, the ADHD group has lower right and left mean caudate volume than the control group in both the manual and automatic analysis. Moreover, the results of the statistical test were the same in the manual and automatic analysis: the volume measure was found to be statistically different between the groups for the right caudate but not for the left. Comparing volume values, it can be seen that the automatic CaudateCut segmentation method under-segments the caudate nucleus compared with the manual delineation. However, these discrepancies in the segmentations do not prevent coherent results between the two methods in the statistical analysis of the groups considered.

**Table 6:** Manual Control and ADHD statistical results

Manual analysis	group	N	M	std	d	t-test	*t*	*p*	CI(95%)
R caudate	Control	39	5031.44	660.18	312.29	**false**	1.9983	0.0493	1.03 to 623.56
	ADHD	39	4719.15	718.81					

L caudate	Control	39	4882.45	643.81	195.11	**true**	1.1946	0.2360	-130.19 to 520.42
	ADHD	39	4687.34	791.17					

Manual analysis of control and ADHD volume statistical differences for right and left caudate volume. N, sample size; M, mean volume (in mm^3^); std, standard deviation, d mean difference; t-test, t-test result; *t*, Student's t statistic; *p*, p-value; CI, confidence interval of mean difference.

**Table 7:** Automatic Control and ADHD statistical results

Automatic analysis	group	N	M	std	d	t-test	*t*	*p*	CI(95%)
R caudate	Control	39	4636.72	596.66	430.19	**false**	2.74	0.0075	118.05 to 742.35
	ADHD	39	4206.52	775.86					

L caudate	Control	39	4426.24	615.69	288.14	**true**	1.93	0.0571	-8.90 to 585.19
	ADHD	39	4138.10	698.89					

Automatic analysis of control and ADHD volume statistical differences for right and left caudate volume. N, sample size; M, mean volume (in mm^3^); std, standard deviation, d mean difference; *t*, Student's t statistic; *p*, p-value; CI, confidence interval of mean difference.

**Figure 8:**
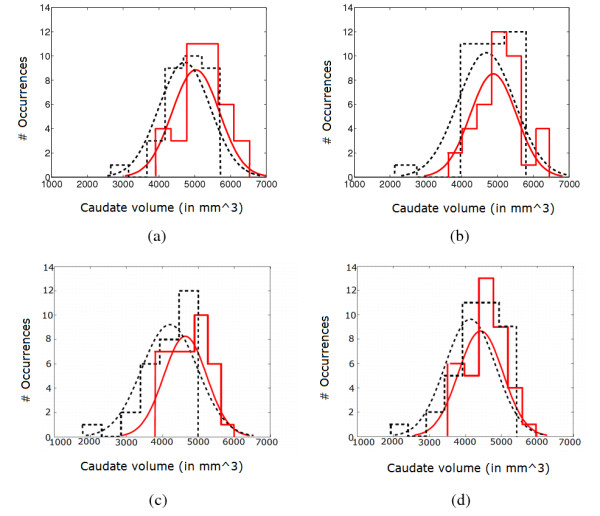
**Control and ADHD caudate volume distributions**. Control and ADHD caudate volume frequency distributions for right and left caudate volumes (in mm^3^). The first row corresponds to manual analysis for right (a) and left (b) caudate nucleus and the second row corresponds to automatic analysis for right (c) and left (d) caudate nucleus. Control (red solid line) and ADHD (black dashed line) histograms are shown together with two Gaussian functions fitted to the histograms.

Finally, we qualitatively compared the manual and CaudateCut automatic analysis. Figure 8 shows both control and ADHD caudate volume distributions using GT segmentation (a, b) and CaudateCut segmentation (c, d). First column plots (a, c) correspond to right caudate volume measures and second column plots (b, d) to left caudate volume measures. The histogram of caudate volume for the ADHD and control groups are depicted in dashed black and solid red lines, respectively. Two Gaussian functions were fitted to the histograms. It can be appreciated that the differences between the ADHD and control distributions were larger for the right caudate in both the manual and the automatic analysis. The immediate conclusion is that CaudateCut generates results that are comparable to gold-standard analyses in differentiating neuroanatomical abnormalities between healthy controls and the group of individuals with ADHD.

## 5 Conclusion

In this work, we present a new method, *CaudateCut*, for caudate nucleus segmentation in brain MRI. CaudateCut combines the power of an atlas-based strategy and the adaptiveness of the defined energy function within the GC energy-minimization framework, in order to segment the small and low-contrast caudate structure. We define the new energy function with data potentials by using intensity and geometry information, and also making the most of the supervised learned local brain structures. Boundary potentials are also redefined using a new multi-scale edgeness measure. CaudateCut has different advantages for different neuroimaging researchers. First of all, it is fully automatic, and secondly, the algorithm is reliable. The results are 100% reproducible in subsequent runs with the same data, avoiding the inaccuracies of intra-rate and inter-rater drift.

The method was tested on two different datasets. Although the method was tuned on the novel URNC database, it provided outstanding results on the IBSR dataset, showing the inherent robustness of the approach. Moreover, we obtained results comparable to manual volumetric analysis of children with ADHD based on automatic caudate nucleus volume measurements. Future lines of research include the use of multiple-hypotheses for seed initialization in order to increase the robustness to possible errors of atlas application and the incorporation of 3D information in the caudate segmentation. From the clinical point of view, new features based on the caudate appearance can be added to analyze ADHD abnormalities in an automatic way.

## Competing interests

The authors declare that they have no competing interests.

## Authors' contributions

LI led this research. She was involved in handling the medical images, the technical novelty of the proposal, its implementation and validation, as well as writing most of this paper. She also supervised and coordinated the team and the different parts of the project. JS was involved in the acquisition of the medical images, the definition of the ground truth, the validation of the method from a clinical point of view, and the writing of the proposal. AH collaborated in the GC part of the technical proposal and its implementation, as well as in the validation and implementation of the comparative method and the writing of the paper. SE collaborated in the GC part of the technical proposal and its implementation, as well as in the validation of the method from a technical point of view and the writing of the paper. XJ collaborated in the atlas-based part of the technical proposal and its implementation, as well as in the validation of the method from a clinical point of view. OV was involved in the acquisition of the medical images, the definition of the ground truth, the validation of the method from a clinical point of view, and the writing of the proposal. PR was involved in supervising the project together with LI, technical discussion of the contribution, validation of the method from both a technical and clinical point of view, and the writing of the proposal. All authors read and approved the final manuscript.

Endnotes

^1^http://www.cabiatl.com/mricro/

^2^http://www.cause07.org/

^3^http://elastix.isi.uu.nl
